# Efficacy and safety of endoscopic ultrasonography-guided interventional treatment for refractory malignant left-sided liver tumors: a case series of 26 patients

**DOI:** 10.1038/srep36098

**Published:** 2016-12-13

**Authors:** Tian-an Jiang, Zhuang Deng, Guo Tian, Qi-yu Zhao, Wei-lin Wang

**Affiliations:** 1 Department of Ultrasound Medicine, the First Affiliated Hospital, Zhejiang University School of Medicine, Hangzhou 310003, China; 2 Department of Hepatobiliary and Pancreatic Surgery, the First Affiliated Hospital, Zhejiang University School of Medicine, Hangzhou 310003, China; 3 Collaborative Innovation Center for Diagnosis and Treatment of Infectious Diseases, Hangzhou 310003, China

## Abstract

This study aimed to compare the efficacy and safety of EUS-guided ethanol injection and ^125^I seed brachytherapy for malignant left-sided liver tumors which were difficult for trans-abdominal intervention. The study protocol was registered at Clinicaltrials.gov (NCT02816944). Twenty-six patients were consecutively and prospectively hospitalized for EUS-guided interventional treatment of refractory malignant left-sided liver tumors between June 2014 and June 2016. Liver masses were detected using EUS in 25 of 26 (96.2%) patients. EUS-guided interventional treatment was completed uneventfully in 23 of 26 (88.5%) patients using anhydrous ethanol injection (n = 10) or iodine-125 seed implantation (n = 13). Six months later, complete response was achieved in 15 of 23 (65.2%) patients and partial response in 8 of 23 (34.8%) patients. Patients with tumor residual have second-look EUS-guided interventional treatment (n = 5), radiotherapy (n = 2) or surgical resection (n = 1). Complete response was achieved after repeated interventional treatment in 3 of 5 patients who underwent second EUS-guided intervention; 2 patients required additional surgical resection but one succeed. No significant complications occurred. Therefore EUS-guided ^125^I seed brachytherapy is an effective and safe treatment modality for radical operation or promising palliative control of malignant left-sided liver tumors refractory to trans-abdominal intervention.

Malignant liver tumors, also known as liver cancer, chiefly consist of primary and metastatic hepatic and intrahepatic bile duct cancers, such as hepatocellular carcinoma, colorectal cancer liver metastasis, and cholangiocarcinoma^1^. Liver cancer, which is usually asymptomatic at an early stage, may be incidentally detected on abdominal imaging scans, including those obtained by ultrasonography, computed tomography (CT), and magnetic resonance imaging (MRI), or may manifest with signs and symptoms including abdominal pain, jaundice, hepatomegaly, abdominal mass, or hepatic dysfunction at a relatively late stage, at which point they are generally associated with a poor prognosis. Radical hepatectomy alone or followed by orthotopic liver transplantation remains the mainstay of treatment for resectable primary liver cancers^2^, while radical resection of liver metastases is also beneficial for eligible colorectal cancer patients after neoadjuvant chemoradiation therapy^3^.

A majority of liver cancer cases are surgically refractory or unresectable possible because of underlying medical conditions, multiple or oversized liver diseases, or involvement of major vessels^4^. Palliative therapy, an alternative to definitive treatment, may help to improve patient survival and quality of life in these settings. Multiple palliative treatment regimens, including transarterial chemoembolization^5^, ablation using anhydrous ethanol injection^6^, high-intensity focused ultrasound^7^ or radiofrequency^8^, iodine-125 brachytherapy^9^, and photodynamic therapy^10^, have been used to control primary and secondary liver cancers. Among these documented palliative treatment modalities, ethanol injection and iodine-125 brachytherapy have shown promising therapeutic effects in liver cancer patients. These two treatment regimens are normally performed percutaneous access. In a few cases, thermal ablation performed in open surgery or under laparoscopy. While percutaneous intervention is generally thought to be minimally invasive, it may not be feasible for some patients with left hepatic lobe atrophy, lesion was located at the surgical margin of the left liver or in the caudate lobe, gastrointestinal flatulence and abdominal skin surgical scar. At this time, the conventional trans-abdominal ultrasound has difficulty to display lesions or even unable to discover it.

Endoscopic ultrasonography (EUS) is an advanced endoscopic technique primarily used for the characterization and biopsy of the gastrointestinal tract, particularly for preoperative staging of malignant diseases, including oesophageal, gastric, and pancreatic cancers^11^. As left lobe of the liver is located in front of the lower esophagus, stomachus cardiacus and in proximity to the gastric lesser curvature, it is accessible on EUS, have better image quality and more adjacent to the focus. Therefore it is possible to interventionally treat left-sided liver tumors through EUS, especially when other percutaneous intervention is not deemed feasible or safe. The present work aimed to compare the efficacy and safety of EUS-guided ethanol injection and ^125^I seed brachytherapy for malignant liver tumors located in the left lobe that are refractory to conventional percutaneous intervention.

## Results

### Demographic and clinical data

**Figure 1:**
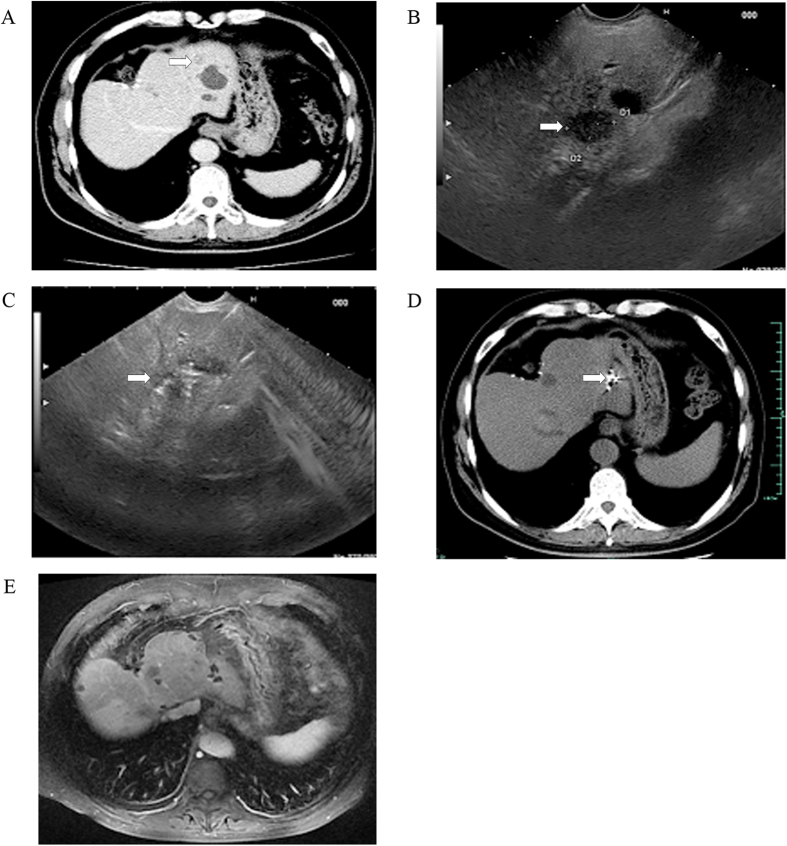
EUS-guided iodine-125 brachytherapy for postoperative liver metastasis of cholangiocarcinoma. (**A**) presence of a 1.3-cm, low-density liver mass located in the left lobe on CT scan (white arrows). US image failed to show the lesions due to the gas ahead; (**B**) identification of a 1.5 cm × 1.3 cm low-echodensity mass on EUS (white arrows); (**C**) EUS-guided implantation of iodine-125 particles (white arrows); (**D**) obvious downsizing of the liver disease on follow-up CT scan at 1 months (white arrows); and (**E**) disappearance of the liver disease on follow-up MRI scan taken after 12 months.

**Figure 2:**
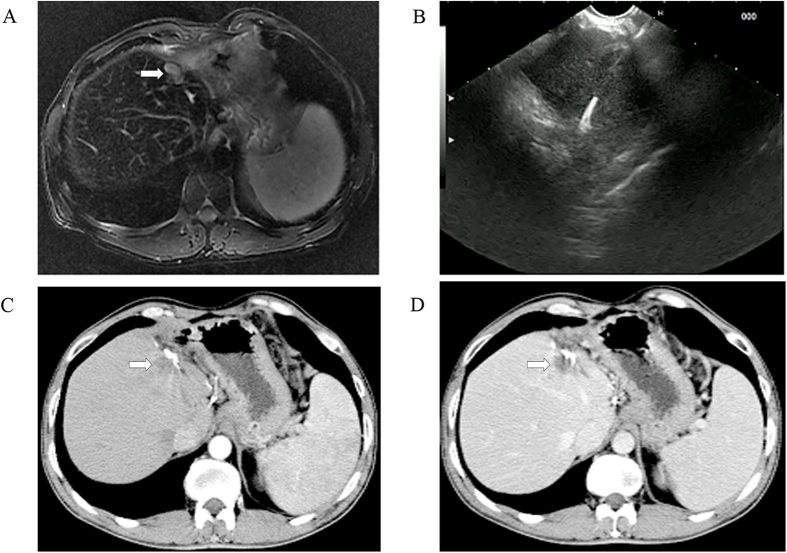
EUS-guided anhydrous ethanol ablation for postoperative liver recurrence of hepatocellular carcinoma. (**A**) presence of a 1.5 cm × 1.1 cm postoperative liver recurrence located on the resection margin as shown on T_2_-weighted MRI scans (white arrows), but conventional US image failed to show the lesions; (**B**) EUS-guided ethanol injection; and (**C**) arterial phase (arrowhead) and (**D**) parenchymal phase (arrowhead): non-enhanced liver disease on follow-up contrast CT scan revealed complete resolution of disease at 12 months.

**Figure 3:**
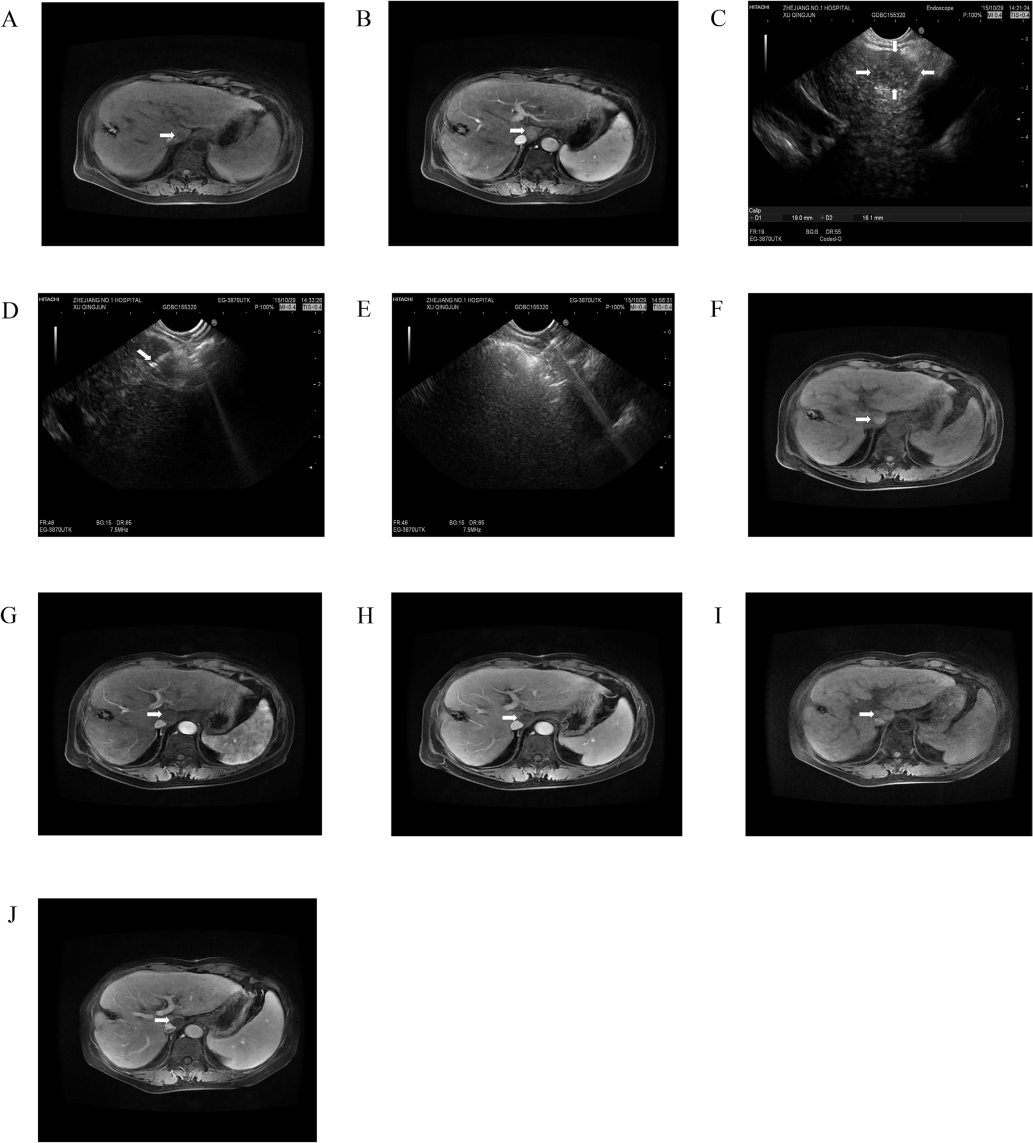
EUS-guided anhydrous ethanol ablation for lesions in liver caudate lobe. MRI imaging in liver caudate lobe showed a lesion of low T1 signal intensity (**A**) (arrowhead) and significant enhancement in arterial phase (**B**) (arrowhead). (**C**) at liver caudate lobe EUS scan showed a well-defined hypoechoic 1.9*1.6 cm lesion (arrows). (**D**) It indicated that 22G biopsy needle with EUS guidance was inserted into the lesion along with the needle sheath (arrowhead), and (**E**) diffuse increase in echogenicity covering the whole mass after the percutaneously punctured injection of anhydrous alcohol. After 1 month follow-up, MR scan was seen high T1-weighted signal intensity (**F**) (arrowhead) and no obvious enhancement during arterial (**G**) (arrowhead) and substance phase (**H**) (arrowhead). After 15 months follow-up, it showed high T1-weighted signal intensity (**I**) (arrowhead) and contrast material-enhanced MR images showed completed ablation without enhanced lesions (**J**) (arrowhead).

Overall, 26 patients were enrolled in this study, including 17 men and 9 women, aged 31–75 years. The basic characteristic of included patients was summarized in Table 1. The aetiologies of the hepatic tumors were chronic hepatitis (n = 6), liver cirrhosis (n = 11), and primary gastrointestinal malignancy (n = 9). Twenty-one patients had a single left-sided liver tumor, and 5 patients had more than one liver tumor with major disease located in the left lobe but multiple tumors in the right lobe. Liver tumors were occurred in left liver shadowed by gastrointestinal (GI) gas (n = 12; Fig. 1), resection margin (n = 7; Fig. 2), caudate lobe (n = 3; Fig. 3), significantly atrophic left lobe (n = 2), and remarkable abdominal skin scar (n = 2). All tumors were not detectable or poorly visualised on percutaneous ultrasound scan because of the above reason. In addition, a systematic review by searching from PubMed up to August 2016 was performed. The summary of 11 studies of ethanol ablation and ^125^I brachytherapy of the hepatocellular carcinoma was presented in Table 2 12,13,14,15,16,17 and Table 3 9,18,19,20,21.

### EUS data

Out of the 26 patients, left-sided liver tumors were detectable in all cases on EUS with the exception of one (1/26, 3.8%) case in which the tumor was located in an atrophic left lobe. Of these 25 cases, 23 were successfully and uneventfully treated using interventional EUS (anhydrous ethanol, n = 10; iodine-125 implant, n = 13) as scheduled. Interventional treatment failed in two patients due to an overly large puncture angulation. Operative time was 10–70 minutes, and no blood loss. No clinically significant procedure-related morbidities occurred.

### Tumor response and mid-term follow-up data

All patients were followed up for 1–2.5 years as scheduled. At 12 months after treatment, 12 of 13 (92.3%) patients achieved complete tumor response in iodine-125 particle implantation (Fig. 1) while 3 of 10 (30%) in anhydrous ethanol ablation (Fig. 3 and Table 4). Residual tumor was detected in 8 patients after anhydrous ethanol ablation (n = 2) or iodine-125 particle implantation (n = 6); these patients were further treated with second-look EUS-guided interventional treatment (n = 5) and radiotherapy (n = 2) and surgical resection (n = 1). Complete response was achieved after again EUS-guided treatment in 3 of 5 patients, whereas the remaining 2 patients required surgical resection but only one succeed. Recurrence was observed in 2 patients at 1 year after anhydrous ethanol ablation. No clinically significant late-onset complications occurred.

## Discussion

Interventional EUS, namely, EUS-guided interventional treatment, has been reported for drainage of pancreatic^22^ or bile duct (pseudo) cysts^23^ or even complex choledochoduodenostomy^24^. This newly emerging interventional treatment incorporating ethanol or chemotherapy injection, brachytherapy, and/or photodynamic therapy has also been piloted in preclinical studies and a small series of clinical studies for the treatment of malignant pancreatic, lower common bile duct, and periampullary diseases^25^. In contrast, EUS is used relatively less frequently for the treatment of hepatic tumors. Awad *et al*.^26^ reported that EUS could detect small-sized primary and secondary liver cancers previously undetectable on CT scans, and simultaneous fine-needle biopsy aided in the diagnosis of deep-seated liver tumors for preoperative staging. Singh *et al*.^27^ also reported, in a small series of prospective patients, that EUS exhibited a higher specificity and positive predictive value than CT and percutaneous ultrasound scanning for metastatic liver tumors located in hepatic segments II and III. Furthermore, interventional EUS has been attempted for transgastric drainage of liver abscess^28^ and infectious biloma after radiofrequency-ablated hepatocellular carcinoma^29^. To the best of our knowledge, the present work is the first prospective study comparing the therapeutic role of EUS-guided ethanol injection and ^125^I seed brachytherapy in the treatment of left-sided liver malignancies.

Percutaneous ultrasonography-guided interventional treatment normally requires an acoustic window to clearly display the liver tumor and allow subsequent ethanol injection, high-intensity focused ultrasound^30^, or other ablation therapy. However, in some patients, such as those enrolled in the current study, hepatic tumors cannot be detected or clearly visualised on percutaneous ultrasound scanning because of distorted liver anatomy from a previous operation, a deep-seated location such as the caudate lobe, at the edge of liver, at the surgical margin that usually affected by gastrointestinal gas or a pre-existing abdominal scar. For left-sided liver tumors, especially when located in proximity to the lesser curvature of the stomach, transgastric EUS can exclude the interference of intestinal gas and provide safe liver access for ethanol injection and iodine-125 implantation. Our results showed that EUS-guided iodine-125 brachytherapy has a better prognosis and less recurrence for left-sided liver tumors with a good safety profile comparing to ethanol ablation. Moreover, iodine-125 implantation is recommended for the treatment of larger tumors because of a higher risk of residual or recurrent tumor. In previous study, it was reported that the 1-, 3- and 5-year recurrence-free rates of the ^125^I brachytherapy after resection of HCC were 94.12%, 76.42%, and 73.65%, and their overall survival rates were 94.12%, 73.53%, and 55.88%, respectively^9^, which was also similar to another study^21^. This may result from that a high-dose short radiation emitted by ^125^I could be continuous within the the target tissues for a long half-life of 59.4 days, which would trigger tumor cell apoptosis and drop proliferation of tumor cells by gathering cells in the radiosensitive cell cycle phase (G2/M)^20^,^31^. The radiation declined sharply with the distance from the seeds and had limited injury to surrounding normal tissues. Meanwhile, ^125^I implantation also could excite the anti-tumor immune response in HCC patients by adding CD3+ and CD4+ immunocytes and facilitating Th2/Th1 deviation^32^. Thus these findings indicated that EUS-guided ^125^I brachytherapy could be a safe and feasible way to effectively target tumor cells and minimize injury to healthy tissues.

There are some limitations regarding EUS-guided interventional treatment of liver cancer. First, interventional EUS requires a more delicate ultrasound system and greater operator expertise; therefore, this interventional technique is less feasible in general ultrasonographic practice. Second, transgastric EUS can only access a relatively small portion of the left lobe; an overly large puncture angulation failed the pre-planned interventional therapy in 2 of our patients. Third, implantation of radioactive particles is relatively complex through a EUS puncture needle compared to the percutaneous approach.

In conclusion, EUS-guided iodine-125 brachytherapy is an effective alternative to ethanol injection for radical operation or promising palliative control of refractory malignant left-sided liver tumors. This technique is minimally invasive and associated with a good safety profile, and residual tumor can be treated by repeated sessions of interventional EUS. The long-term efficacy and safety of interventional EUS have yet to be investigated in large-scale, comparative studies, especially studies evaluating patients’ survival and quality of life.

## Patients and Methods

### Patient selection

Between June 2014 and June 2016, 26 patients with primary or metastatic left-sided liver tumors diagnosed on abdominal ultrasound, CT, and/or MRI scans, who were ineligible for surgical treatment due to unresectable disease or high risk of surgical morbidity, were consecutively and prospectively referred to our ultrasound centre for palliative treatment. The included patients were randomly divided into two groups: 10 in the anhydrous ethanol group and 13 in the iodine-125 implantation group. Inclusion criteria were as follows: American Society of Anaesthesiologists physical status class I and II; relatively well confined, single hepatic tumor or extensive hepatic tumor (in the scenario of multiple foci) located in Couinaud segments I–III refractory to percutaneous access; liver tumor that could not be visualised or not clearly visualised on percutaneous ultrasound; and generally normal or compensated liver function (Child-Pugh classes A and B). Exclusion criteria were as follows: a known history of or suspected upper gastrointestinal stenosis or stricture; complicating oesophagogastric varices or bleeding; pre-existing serious cardiopulmonary or hepatorenal functional impairment or coagulopathy; extensive intra- or extrahepatic metastases; or refusal to participate in the study. All patients provided informed consent in writing prior to enrolment.

### Ethical issues

This was designed as a randomized, open-label, control study. The clinical trial was registered in Clinicaltrials.gov ID: NCT02816944 on June 23th 2016. All patients and relatives were informed about the purpose and procedures of this study and gave written informed consent. Eligible patients were randomly allocated into the ^125^I brachytherapy group and the control group of ethanol ablation. The study was approved by the Institutional Review Board at the First Affiliated Hospital of Zhejiang University School of Medicine, Hangzhou and all studies were conducted in accordance with relevant guidelines and regulations.

### EUS and interventional treatment procedures

All patients underwent routine hematologic, clinical biochemical, coagulative function, virologic, and oncologic assays prior to interventional EUS. All interventional procedures were performed by an assigned interventional treatment team led by a board-certified interventional physician, including resident interventional physicians, radiologists, and research nurses.

Left-sided liver EUS was performed as previously described^28^. Briefly, the patient was placed in the left recumbent position and sedated using intravenous propofol. After excluding the presence of oesophagogastricvarices using conventional endoscopy, a Pentax endoscopic ultrasound system (Pentax Medical, Tokyo, Japan) incorporating a Hitachi colour Doppler ultrasound scanner (Hitachi Medical, Tokyo, Japan) was used for EUS of the left-sided liver. An endoscopic ultrasound injection needle (Cook Medical, Bloomington, IN, USA) was used for injection of anhydrous ethanol (22G) for well-encapsulated primary hepatocellular carcinoma or implantation of iodine-125 particles (19G; Cook Medical, Bloomington, IN, USA) for hepatocellular carcinoma, cholangiocellular carcinoma or metastatic liver cancer.

### Postoperative care, follow-up procedure, and main outcome measures

Patients were closely followed after EUS and treated with antimicrobial, haemostatic, and hepatoprotective agents as indicated. All patients were followed up at the outpatient clinic using abdominal ultrasound, CT, and/or MRI at 1, 3, 6, and 12 months postoperatively. The main outcome measures were frequency of detection by EUS, tumor response as defined by *the Response Evaluation Criteria in Solid Tumors*^30^ at 6 months, and interventional EUS-associated morbidities. In this study, all statistical analysis was performed using x^2^, Fisher exact test and Student t test by spss 13.0 software.

## Additional Information

**How to cite this article**: Jiang, T.-a *et al*. Efficacy and safety of endoscopic ultrasonography-guided interventional treatment for refractory malignant left-sided liver tumors: a case series of 26 patients. *Sci. Rep.* **6**, 36098; doi: 10.1038/srep36098 (2016).

**Publisher's note:** Springer Nature remains neutral with regard to jurisdictional claims in published maps and institutional affiliations.

## Figures and Tables

**Table 1:** Demographic characteristics of the patients.

Item	^125^I	EI	Statistical value	*P* value
No. Cases	13	10		
Gender			NA^#^	0.197
male	6	8		
female	7	2		
Age	55.5 ± 7.6	56.2 ± 3.1	t = −0.176	0.862
Maximum diameter	2.8 ± 1.2	2.3 ± 0.6	t = −1.229	0.235
Histoty of disease			x^2^ = 0.619	0.734
chronic hepatitis	3	3		
liver cirrhosis	4	4		
primary gastrointestinal malignancy	6	3		
Number of tumors				
single	12	6	NA^#^	0.343
multiple	2	3		
Recurrence	0	2	NA	NA

^#^means the result was calculated by Fisher’s exact test; EI: ethanol injection; NA: not available.

**Table 2:** Summary of ethanol injection for treatment of hepatocellular carcinoma.

Author	Year	Country	Characteristics of patients	Treatment method	Patients(No. of tumors)	Tumor size	Male/female	Mean age(range)	Follow-up interval(months)	Prognosis	Complication
Nakaji S *et al*.	2015	Japan	Patient with HCC in the left hepatic lobe	Eus-guided EI	1	45	0/1	79	1	CR	No
Nakaji S *et al*.	2012	Japan	Patient with HCC	Eus-guided EI	1(2)	13 and 17	1	82	0.5	CR	NA
Azab M *et al*.	2011	Egypt	Patient with HCC	US-guided PEI	32	NA	NA	46–77	18	24 CR;8 PR	25 transient pain;2 portal vein thrombosis;3 fever;3 mild ascites
Giorgio A *et al*.	2011	Italy	Patient with HCC	US-guided PEI	142	23.4 ± 4.5(11–30)	105/37	70 ± 2(68–74)	22	3- and 5-year survival rates:74% and 68%;3- and 5-year local recurrence rates were 9.4% and 12.8%	Major complications was 1.9%
Brunello F *et al*.	2008	Italy	Patient with HCC	US-guided PEI	69 (88)	22.5 ± 5.4	49/20	70.3 ± 8.1	25.3	1-year OS rate 83.3%	1 haemoperitoneum;1 death
Shiina S *et al*.	2005	Japan	Patient with HCC	US-guided PEI	114 (192)	45 ≤2 cm; 73 >2 cm	87/27	41 ≤65;73 >65	34.8(1.2–50.4)	a) Solitary tumor: 1-, 3- and 4-year survival rates:95%, 85%, 73%, and 64%; b) multiple tumors:1-, 3- and 4-year survival rates:93%, 78%, 60%, and 48%	1 liver abscess;2 neoplastic seeding

HCC: hepatic cellular cancer; US: ultrasound; EI: ethanol injection; PEI: percutaneous ethanol injection; NA: not available; CR: complete response; PR: partial response; OS: overall survival.

**Table 3:** Summary of ^125^I brachytherapy for treatment of hepatocellular carcinoma.

Author	Year	Country	Characteristics of patients	Treatment method	Patients(No. of tumors)	Tumor size	Number of seeds	Male/female	Mean age(range)	Follow-up interval(months)	Prognosis	Complication
Zhang W *et al*.	2015	China	1 patient with Bismuth type IV Klatskin tumor	DSA	1	NA	12	1	75	3	Improved	NA
Chen K *et al*.	2013	China	Patients with hepatocellular carcinoma after complete hepatectomy	^125^I brachytherapy	34(38)	6.24 ± 2.55	25(18–34)	25/9	18–70	47.6 (7.7–106.4)	Time to recurrence:60 months;1-, 3- and 5-year recurrence-free rates:94.12%, 76.42%, and 73.65%;1-, 3- and 5-year OS rates:94.12%, 73.53%, and 55.88%.	1 nausea;1 sinus tachycardia;1 premature atrial contraction
Luo J *et al*.	2011	China	Patients with HCC complicated by tumor thrombus in main portal vein	US-guided ^125^I seed strand and stent with chemoembolization	32	87.6 ± 28.2 (53–175)	17	28/4	53.2 ± 8.8 (30–76)	7.3 ± 5.1	Technical success rate was 100%; the 3-month, 6-month, and 12-month cumulative survival rates were 96.4%, 67.4%, and 39.3%, and the cumulative stent patency rates were 96.7%, 83.4%, and 83.4%	Fever, vomiting or upper abdominal pain
Zhang FJ *et al*.	2008	China	Patients with HCC of PVTT	US-guided 125I brachytherapy	19	11 ≥5 cm; 8 <5 cm	18–30	13/6	57 (37–68)	3–22	12 CR;4 PR; 3 stable	Mild pain;1 hemothorax
Nag S *et al*.	2006	America	Patients with unresectable or residual disease after surgical resection	^125^I brachytherapy	64(309)	NA	40(10–134)	33/31	57.4 (30–81)	158.4 (20–175)	Median time to recurrence:9 months (6–12 months);1-, 3- and 5-year intrahepatic local control rates were 44%, 22%, and 22%;1-, 3- and 5-year OS rates were 73%, 23%, and 5%	2 died;1 small-bowel obstruction;1 small-bowel perforation;1 liver abscess;1 wound abscess.

HCC: hepatic cellular cancer; PVTT: portal vein tumor thrombosis; DSA:digital subtraction angiography; US: ultrasound; NA: not available; CR: complete response; PR: partial response; OS: overall survival.

**Table 4:** Result of the patients in anhydrous ethanol injection group and iodine-125 seed implantation group.

Item	^125^I	EI	Statistical value	*P* value
CR	12	3	NA^#^	0.006
PR	1	7		

^#^means the result was calculated by Fisher’s exact test; EI: ethanol injection; CR: complete response; PR: partial response; NA: not available.
